# Aid or Antagonize: Nuclear Long Noncoding RNAs Regulate Host Responses and Outcomes of Viral Infections

**DOI:** 10.3390/cells12070987

**Published:** 2023-03-23

**Authors:** Viraj Kulkarni, Sahana Jayakumar, Mahesh Mohan, Smita Kulkarni

**Affiliations:** 1 Disease Intervention and Prevention Program, Texas Biomedical Research Institute, San Antonio, TX 78227, USA; vkulkarni@txbiomed.org; 2 Host-Pathogen Interaction Program, Texas Biomedical Research Institute, San Antonio, TX 78227, USA; sjayakumar@txbiomed.org (S.J.); mmohan@txbiomed.org (M.M.)

**Keywords:** lncRNA, virus, epigenetics

## Abstract

Long noncoding RNAs (lncRNAs) are transcripts measuring >200 bp in length and devoid of protein-coding potential. LncRNAs exceed the number of protein-coding mRNAs and regulate cellular, developmental, and immune pathways through diverse molecular mechanisms. In recent years, lncRNAs have emerged as epigenetic regulators with prominent roles in health and disease. Many lncRNAs, either host or virus-encoded, have been implicated in critical cellular defense processes, such as cytokine and antiviral gene expression, the regulation of cell signaling pathways, and the activation of transcription factors. In addition, cellular and viral lncRNAs regulate virus gene expression. Viral infections and associated immune responses alter the expression of host lncRNAs regulating immune responses, host metabolism, and viral replication. The influence of lncRNAs on the pathogenesis and outcomes of viral infections is being widely explored because virus-induced lncRNAs can serve as diagnostic and therapeutic targets. Future studies should focus on thoroughly characterizing lncRNA expressions in virus-infected primary cells, investigating their role in disease prognosis, and developing biologically relevant animal or organoid models to determine their suitability for specific therapeutic targeting. Many cellular and viral lncRNAs localize in the nucleus and epigenetically modulate viral transcription, latency, and host responses to infection. In this review, we provide an overview of the role of nuclear lncRNAs in the pathogenesis and outcomes of viral infections, such as the Influenza A virus, Sendai Virus, Respiratory Syncytial Virus, Hepatitis C virus, Human Immunodeficiency Virus, and Herpes Simplex Virus. We also address significant advances and barriers in characterizing lncRNA function and explore the potential of lncRNAs as therapeutic targets.

## 1. Introduction

During the past decade, several genome-wide RNAi [1,2,3,4,5,6,7,8,9,10,11,12,13,14] and CRISPR [15,16,17,18,19,20,21,22] studies have identified host proteins critical for viral replication. Protein-coding open reading frames constitute less than 2% of the human genome [23], and the bulk of the transcriptome is noncoding RNA (ncRNA). Among the different ncRNA subclasses, long noncoding RNAs (lncRNAs) were reported to constitute nearly 68% of the transcriptome [24]. Many lncRNAs regulate various cellular processes [25,26] and are emerging as versatile regulators of gene expression with prominent roles in health and disease [27,28].

A variety of viral infections alter host lncRNA expressions. Dramatic changes in lncRNA expressions have been observed in the cells infected with Influenza A Virus (IAV) [29], Sendai Virus (Sev), Respiratory Syncytial Virus (RSV) [30], Hepatitis C Virus (HCV) [31], Adenovirus [32], Human Papilloma Virus (HPV) [33], pathogenic human enterovirus [34], Severe Acute Respiratory Syndrome Coronavirus (SARS-CoV and SARS-CoV-2) [35,36,37,38,39,40,41,42,43,44,45], Human Immunodeficiency Virus (HIV) [46,47,48,49,50,51], Muscovy Duck ReoVirus (MDRV), and Herpes Simplex Virus (HSV) [52]. These studies are only beginning to reveal the myriad changes in lncRNA expression upon viral infection and indicate the role of lncRNAs in viral pathogenesis.

The pathogen-associated molecular patterns (PAMPs) and damage-associated molecular patterns (DAMPs) released during infections, stress, and non-programmed cell death are detected by the pattern recognition receptors (PRRs), such as Toll-like receptors (TLR), retinoic acid-inducible gene (RIG)-like receptors (RLRs), nucleotide oligomerization domain (NOD)-like Receptors (NLRs), and C-type lectin receptors (CLRs). The activation of PRRs leads to the transcription of inflammatory genes induced by ATF2 and NF-κB, the transcription of specific antiviral genes induced by IRF3 and IRF7, and the synthesis of Type I interferons (IFN-I). PRR activation by virus infection [53,54,55,56,57,58] and various ligands, such as lipopolysaccharides (TLR4) or Poly I: C induces the expression of lncRNAs [59,60,61,62,63,64]. In addition, stimulating cells by cytokines, such as IFN-I [65] and TNF-α [66], induces differential expressions of lncRNAs. Viral infections, including specific viral proteins can upregulate the expression of stress-induced and other lncRNAs [67,68]. Thus, the transcriptome of virus-infected cells presents an opportunity to discover and characterize novel lncRNAs that may play a significant role in cellular defense, immune response, and viral propagation. For instance, IFN–alpha (IFNα) stimulation or infection with RNA viruses upregulates lncRNA *ISIR* [69]. *ISIR* activates the Interferon Regulatory Factor-3 (IRF3) and strengthens the interferon response to viral infections. Indeed, several transcriptomic studies have highlighted the marked dysregulation of lncRNAs in virally infected cells [70]. We have listed prominent nuclear RNAs with a significant impact on immune response, viral replication, and latency in Table 1. We also discuss the molecular mechanisms of their action wih more details in this review.

Overlapping patterns in lncRNA expression, in response to virus infections, suggest the functional role of lncRNAs in the clinical manifestations of these infections. Similarly, intersecting changes in global lncRNA expression patterns in SARS-CoV and influenza virus infection indicate a lncRNA-based signature of respiratory virus infection and a functional role for the virus-induced lncRNAs in clinical outcomes [95]. After performing a whole-transcriptome analysis of the host response to severe acute respiratory syndrome coronavirus (SARS-CoV) infection across four founder mouse strains, Peng et al. found several noncoding RNAs to be similarly regulated in SARS-CoV and influenza virus-infected mice [95]. However, the functional mechanisms and impact of these overlapping patterns of lncRNAs in respiratory viral infections are yet to be determined. In addition, the virus-induced lncRNAs may have diagnostic potential, such as the antiviral lncRNA EDAL induced by multiple neurotropic viruses in mice [96]. Thus, careful analyses of lncRNA expression and function in infected cells are critical to improving our understanding of viral pathogenesis.

Nevertheless, diversity in the form and function of lncRNAs makes them both intriguing and challenging to study. The human DNA is predicted to encode over 100,000 lncRNAs [97,98], but only a tiny fraction of these have been characterized. Several types of lncRNAs are described in the literature, including but not limited to lncRNAs transcribed from intergenic, enhancer, and promoter regions, as well as sense and antisense lncRNAs that overlap other protein-coding genes [23,99,100]. Most lncRNAs are expressed at lower levels than protein-coding mRNAs. Several factors, including repressive histone modifications at lncRNA gene promoters [101,102], transcription through phosphorylation-deficient Polymerase II (pol II), weak or aberrant splicing, and termination contribute to lower transcription levels of lncRNAs than the protein-coding mRNAs. Additionally, degradation by nuclear exosomes leads to overall diminished expression levels of most lncRNAs [103]. Nevertheless, the expression levels of several lncRNAs are either similar or even exceed those of protein-coding mRNAs. For example, *MALAT1* and *NEAT1* are expressed ubiquitously at high levels in most cells [104].

A significant fraction of lncRNA is preferentially localized in the nucleus. This nuclear retention is due to specific sequence motifs encoded within some lncRNAs, such as Alu repeats [105], AGCCC motif of *BORG* [106], E and M fragments of *MALAT1* [107], repeating RNA domain (RDD) of human *FIRRE* [108], and retained introns in the nuclear *TUG1* [109]. The nuclear retention of Kaposi Sarcoma-associated Herpes Virus (KSHV)-encoded lncRNA and *PAN RNA* (polyadenylated nuclear RNA) is dependent on the presence of an expression and nuclear retention element (ENE) [110]. The ENE contains a uracil (U)-rich internal loop that interacts with the 3′-poly(A) tail to form a triple helical loop to protect *PAN* RNA from a rapid nuclear deadenylation-dependent decay by exonucleases [111,112,113]. In addition, viral ORF57 stabilizes the expression of *PAN* RNA and increases its nuclear accumulation [88,114,115]. RNA-stabilizing ENE-like structures are also found in human lncRNAs, such as *MALAT1* and *MENβ* [116]. Protein-coding mRNAs and many lncRNAs share the mechanisms for posttranscriptional processing, nuclear export, and trafficking within the cells [117]. Like the protein-coding mRNAs, many lncRNAs are exported from the nucleus by the nuclear export complexes, TREX (transcription export complex), and NFX (nuclear RNA export factor) [117,118,119]. However, some lncRNAs are retained in the nucleus due to poor binding to the nuclear export complexes [119,120]. In addition, functional nuclear lncRNAs escape exosome degradation through specific, high-affinity interactions with DNA and proteins that tether them to the chromatin.

Functional nuclear lncRNA transcripts employ diverse mechanisms to regulate gene expression, including but not limited to the recruitment, depletion, or relocalization of chromatin-modifying proteins, transcription factors, and RNA; the direct interaction with DNA; and the regulation of chromatin organization, as well as intra/inter-chromosomal interactions, transcription, and splicing [108,121,122,123,124,125,126,127]. Nuclear lncRNAs have been shown to regulate the localization of splicing factors and impact the expression of antiviral genes [83,128]. LncRNAs are typically expressed at significantly lower levels than protein-coding mRNAs. Per-cell copies of lncRNAs (0.3–1000) are remarkably low compared to their protein binding partners. Recent data show that lncRNAs adopt distinct mechanisms to affect dramatic changes in target gene expression, even with a few copies. A few lncRNA molecules seed and organize functional territory wherein the lncRNAs recruit diffusible RNAs or proteins, thus enriching the functional effectors at a specific genomic site [129,130,131]. Quinodoz et al. showed that most lncRNAs included in their study remain at their target loci close to the lncRNA transcription site and do not diffuse elsewhere in the nucleus or cytoplasm [130]. Markaki et al. further elucidated how only a few lncRNA molecules can initiate the “crowding” of transcriptional regulators at several target loci [129]. *Xist* lncRNA silences over 1000 genes on the X-chromosome. Markaki et al. showed that just two *Xist* RNA molecules could recruit a multiprotein structure and increase the local concentration of regulators to silence the transcription of target genes. X-chromosome compaction and densification of a silencer protein, SPEN, induces silencing across the entire X-chromosome. Thus, X-chromosome genes that do not directly interact with *Xist* RNA are also silenced [129]. Markaki et al. also showed that *Xist* remains restricted on the X-chromosome [129]. Other lncRNAs, such as *MALAT1,* interact with several genes far from their transcription site and across chromosomes. These observations raise further questions about how some lncRNAs stay localized to their transcription site, and if any specific features dictate lncRNA movement and localization. For some lncRNA loci, the transcript does not exhibit any regulatory function. However, the process of lncRNA transcription itself may contribute to the regulation of adjacent gene expression by remodeling chromatin or recruiting transcriptional regulatory factors [132]. Thus, nuclear lncRNAs are versatile tools for rapidly activating or suppressing specific genes or gene networks, and some viruses have been shown to hijack this machinery to establish infections successfully.

Here, we review the impact of nuclear lncRNAs on the regulation of gene expression and viral disease outcomes. Most nuclear lncRNAs described here utilize epigenetic mechanisms to regulate gene transcription. Nevertheless, we also have a few nuclear lncRNAs that play a significant role in viral infections by regulating mRNA splicing of the host response genes.

## 2. Chromatin–lncRNA Interactions Regulate Viral Infections

Chromatin structure plays a critical role in activating and repressing transcription. Many lncRNAs modulate gene expression within the spatial proximity of their transcription site and distant gene networks through either sequence-specific, direct DNA-binding, or indirectly through their chromatin-binding protein partners. Emerging data indicate that lncRNA-chromatin interactions regulate the antiviral interferon (IFN) response, viral transcription, and latency. We have listed several lncRNAs with known antiviral or proviral activity in Table 1. The precise mechanism of action of some lncRNAs remains to be further explored. This section discusses known nuclear lncRNAs regulating immune response and viral transcription through direct interaction with chromatin and histone modification.

**Figure 1:**
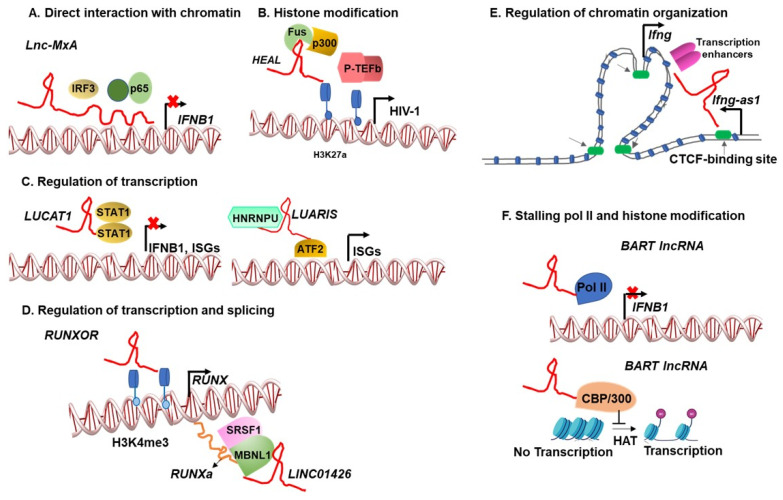
Virus-induced nuclear lncRNAs regulate gene expression using various mechanisms, such as (**A**) Direct interaction of *Lnc-MxA* with the promoter region of *IFNB1,* which inhibits recruitment of transcription factors and *IFNB1* transcription; (**B**) *HEAL* lncRNA recruits histone-modifying proteins and transcription elongation factors at HIV promoter and enhances HIV transcription; (**C**) *LUCAT1* sequesters STAT1 transcription factor and inhibits *IFNB1* transcription, whereas *LUARIS* localizes ATF2 to the promoter region of interferon-stimulated genes (ISGs) and enhances their expression; (**D**) *RUNX* mRNA transcription and isoform expression are regulated by neighboring lncRNAs *RUNXOR* and *LINC01426*; (**E**) *Ifng-as1* transcript is likely to enhance *Ifng* expression by recruiting and enriching transcriptional enhancers (transcription factor or chromatin modifiers). In addition, the *Ifng-as1* locus impacts the chromatin organization independent of the *Ifng-as1* transcription or lncRNA sequence through a CTCF-binding site encoded within the *Ifng-as1* gene region; (**F**) *BART* lncRNA stalls Pol II at the promoter region of *IFNB1* and inhibits its transcription. *BART lncRNA* also associates with CREB-binding protein (CBP/300) and inhibits its histone acetylation activity, thus inhibiting gene expression.

Direct interaction: Many nuclear lncRNAs interact with the double-stranded DNA directly in a sequence-specific manner. These interactions form triple helices [133,134,135,136,137,138,139,140] and R-loops [141]. The triple helices recruit coactivator or corepressor proteins to activate [134,136,139] or repress [133,135] gene transcription, respectively. The lncRNA–DNA triple helices can form near the transcription start site (proximal) or at distant regulatory regions (distal). *Lnc-MxA* is an IFN-induced lncRNA upregulated during IAV infection. *Lnc-MxA* binds to the *IFNB1* promoter forming a triplex, which then interferes with the binding of IRF3 and p65 transcription factors to the *IFNB1* promoter, resulting in the abrogation of the *IFNβ* transcription [61] (Figure 1). Type I IFNs, such as IFNβ, promote the transcriptional activation of hundreds of interferon-stimulated genes (ISGs), many of which inhibit virus replication [142]. Thus, *Lnc-MXA* enhances viral replication by dampening the interferon response [61].

Elevated expression levels of lncRNA *MIR4435-2HG* have been reported in primary myeloid-derived dendritic cells (mDCs) isolated from patients who spontaneously controlled HIV replication (elite controllers, ECs) [134]. An elevated expression of *MIR4435-2HG* increased triple-helix formations at an intronic gene enhancer and enhanced *RPTOR1* (Regulatory Associated Protein Of MTOR Complex I) gene expression. Activating chromatin marker H3K27ac is enriched at the site of the triple helix, likely through the specific recruitment of histone acetyltransferases [134]. RPTOR1 increased glycolysis and metabolic activity of mDCs in response to TLR3 stimulation. Hartana et al. identified a role for *MIR4435-2HG* in enhancing the metabolic activity of mDCs, which is likely to increase the functional responsiveness of mDCs, thereby facilitating more effective immune activity in ECs [134].

Virally encoded lncRNAs also use this mechanism to modulate viral gene transcription. For example, the Epstein–Barr virus (EBV) forms virus-induced nodular structures (VINORCS). VINORCS are composed of viral and cellular proteins required for viral replication. EBV-encoded lncRNA *BHLF1* localizes in the viral replication compartment [93], forming an RNA–DNA hybrid at the virus transcription start site [94]. This hybrid structure then recruits RNA-binding proteins to form VINORCs and facilitate selective processing and the export of viral mRNAs, thus enhancing viral replication.

Histone modification: Post-translational histone modifications, such as phosphorylation, acetylation, methylation, ubiquitination, SUMOylation, and GlcNAcylation, are key regulators of the chromatin state and transcriptional activity. Nuclear lncRNAs interact with the proteins that add (writers) [143,144,145], remove (erasers) [144,146,147], or recognize (readers) [148,149] these histone modifications and modulate their functions. Several lncRNAs use this mechanism to regulate the expression of ISGs. The expression of some ISG-regulating lncRNAs is significantly modulated in virus-infected cells. For example, viruses such as IAV, SeV, MDRV, and HSV significantly downregulate lncRNA *NRAV*, which inhibits the expression of critical ISGs through histone modifications at the promoters of these genes. In this context, an *NRAV* overexpression leads to reduced H3K4 trimethylation (H3K4me3), activating transcription and enriched repressive H3K27 trimethylation (H3K27me3) at the transcription start sites of ISGs [57]. Although the exact mechanism of how *NRAV* regulates histone trimethylation is unknown, *NRAV* is shown to bind a regulatory protein ZONAB, which may be involved in histone modifications. The *NRAV*-mediated regulation of ISG transcription was attributed, at least partially, to its interaction with ZONAB [57]. Likewise, a host cell-encoded lncRNA induced in response to IAV infection, termed, “Inhibiting IAV Replication by Promoting IFN and ISG Expression” (*IVRPIE*), enhances the expression of IFN-β and several critical ISGs, including IRF1, IFIT1, IFIT3, MxA, ISG15, and IFI44L, through histone modifications at these loci [63]. MxA and ISG15 can suppress the replication of highly pathogenic Influenza A viruses [150]. Likewise, IFITM1 and IFITM3 can inhibit an early step of Influenza A virus replication [2]. Thus, *IVRPI* inhibits IAV replication by enhancing the expression of IFN-β and antiviral ISG proteins. HCV-induced lncRNA *RP11-288L9.4* inhibits the expression of IFNα-inducible Protein 6 (IFI6) through histone modifications [72].

The host-encoded lncRNA *HEAL* is expressed at high levels in human macrophages upon HIV infection and binds directly to the HIV promoter, along with an RNA-binding protein fused in liposarcoma (FUS) [85]. The *HEAL*–FUS complex recruits the histone acetyltransferase p300 to enhance H3K27 acetylation and enriches Transcription Elongation Factor P-TEFb to the HIV promoter, thus increasing HIV transcription [85] (schematic presentation of *HEAL*–FUS-driven HIV transcription in Figure 1). LncRNA *MALAT1* interacts with Polycomb Repressive Complex 2 (PRC2) proteins, namely EZH2, Suz12, and EED, which then catalyze the methylation of Histone H3 at Lysine 27 to repress gene transcription. In tumor cells, *MALAT1* facilitates EZH2-binding to its target loci, driving H3K27 trimethylation-mediated repression of multiple tumor-suppressor genes, such as E-cadherin [151,152], NDRG1 [153], p21, and p27 [154]. *MALAT1* is expressed at high levels in HIV-infected cells, where it enhances HIV transcription from latent provirus [78]; it localizes EZH2 to its target sites in tumors; and in HIV infection, it sequesters EZH2 away from the HIV long terminal repeat (LTR), thus preventing PRC2-mediated H3K27 trimethylation and promoting HIV viral reactivation [78].

The KSHV-lytic protein (K-Rta) induces the expression of a cellular lncRNA *KIKAT/LINC01061* (KSHV-Induced KDM4A-Associated Transcript) [68]. Yang et al. showed that *KIKAT* interacts with a histone lysine demethylase (KDM4A) and re-localizes KDM4A from the transcription start site (TSS) of the Angiomotin (AMOT) gene. The *KIKAT*-mediated relocation of KDM4A increased the AMOT transcription and angiomotin-dependent cell migration, thus implicating its role in angiogenesis in Kaposi’s sarcoma [68]. *KIKAT* was also shown to enhance KSHV reactivation. While KDM4A was shown to regulate KSHV replication [155,156], its binding on the KSHV genome was not impacted by *KIKAT* [68].

Virus-encoded lncRNAs also utilize histone modification strategies to establish and regulate their latent infection. This mechanism has been demonstrated in HIV infection, where a virus-encoded antisense lncRNA (antisense transcript; *Ast*) recruits chromatin remodeling proteins such as DNMT3a, EZH2, and HDAC-1 to HIV 5’ long terminal repeat (LTR). These proteins mediate H3K9 dimethylation, H3K27 trimethylation, and histone deacetylation, resulting in the epigenetic silencing of viral transcription [86,157,158].

KSHV remains persistent for a lifetime in patients. KSHV remains latent for a long duration, followed by a short lytic cycle. During latency, the KSHV episome is tethered to the host genome through KSHV latency-associated nuclear antigen (LANA) protein (KSHV life cycle is reviewed in [159]). LANA protein binds the viral episome and the host nucleosomal proteins to tether the viral episome to the cellular genome. The KSHV-encoded lncRNA, *PAN* RNA ([160], is the most abundant viral transcript [114]. The absence of *PAN* RNA results in reduced virus production [88,161,162]. The KSHV-lytic protein (K-Rta) induces *PAN* RNA expression at very high levels [163]. *PAN* RNA interacts with KSHV latency-associated nuclear antigen (LANA) and sequesters LANA from the viral DNA episomes, thus facilitating lytic reactivation [114,164]. A recent study showed that *PAN* RNA sequesters LANA-interacting nucleosomal protein CHD4 (chromodoain helicase binding protein 4) [165]. The CHD4 and LANA complex co-localize to the episome, where CHD4 prevents the aggregation of RNA polymerase II on the KSHV episome and inhibits KSHV reactivation [165]. Thus, *PAN* RNA facilitates KSHV reactivation by the sequestration of the LANA/CHD4 complex from the KSHV episome [165]. *PAN* RNA encodes two main *cis*-acting elements, the Mta response element, (MRE) and the expression and nuclear retention element (ENE). Gutierrez et al. found that ENE is not required for viral replication but is essential for the nuclear retention of *PAN* RNA [110]. Viral protein ORF59 binds to *PAN* RNA during reactivation, recruiting chromatin-modifying factors to the viral genome [166]. *PAN* RNA physically interacts with the viral promoter, lysine demethylases UTX and JMJD3, and lysine methyltransferase MLL2 [87]. It recruits histone demethylases to the viral genome [88]. In addition, *PAN RNA* interaction sites have also been detected on the host genome, suggesting that it potentially interacts with transcriptional regulators and chromatin modifiers to modulate cellular gene expression, immune response, and cell cycle control [88,162].

## 3. Virus-Induced lncRNAs Regulate the Transcription and Splicing of Host and Viral Genes

Virus-induced lncRNAs modulate antiviral interferon responses by regulating the activation, availability, and localization of transcription factors. *Lnc-000641*, a pseudorabies virus (PRV)-induced lncRNA, inhibits the phosphorylation of upstream-activating kinases and transcription factors (Jak and STAT1), thereby reducing downstream IFNα transcription, thus facilitating increased PRV replication [73]. Along similar lines, HCV-induced lncRNA, *Lethe*, interacts with NF-κB subunit RelA and inhibits RelA-mediated DNA binding [66]. This prevents RelA-mediated transcriptions of the activating antiviral factors, 2′,5′-oligoadenylate synthetase (OAS), interferon regulatory factor 1 (IRF1), and protein kinase R (PKR), thus, enhancing HCV replication [71]. LPS-stimulated or virus-infected human dendritic cells (DCs) upregulate the expression of lncRNA *LUCAT1*, which functions as a potent regulator of the IFN-α/β response [167]. *LUCAT1* sequesters STAT1 in the nucleus preventing STAT1 from binding to the promoters of ISGs and blocking their expression [167] (Figure 1). The virus infection or activation through PAMPs can also downregulate the expression of lncRNAs that regulate the immune response. For example, Aznaourova et al. recently showed that SARS-CoV-2 infection or PAMP-mediated stimulation inhibits the expression of a nuclear lncRNA, *PIRAT*. *PIRAT* recruits transcription factor PU.1 to pseudogenes and suppresses PU.1 binding to promoters of alarmin genes (*S100A8* and *S100A*), thus inhibiting alarmin gene expression. Alarmins promote the production of inflammatory cytokines. The SARS-CoV-2 infection also enhances the expression of another lncRNA, *LUCAT1*, which augments alarmin gene transcription. Thus, the SARS-CoV-2 infection upregulates *LUCAT1* and downregulates *PIRAT*, increasing alarmin production and aggravating inflammatory mediators contributing to the severity of COVID-19 [168]. LncRNA *LUARIS* (a.k.a. *lncRNA#32*) upregulated the ISG expression through interactions with host proteins HNRNPU and ATF2, resulting in the inhibition of encephalomyocarditis virus (EMCV), Hepatitis B, and Hepatitis C virus replication [76]. While HNRNPU stabilized *LUARIS* transcript, the *LUARIS*–ATF2 interaction was found to be critical for activating ISG transcription, indicating that *LUARIS* enhanced recruitment of the transcription factor ATF2 to the promoters of ISGs, thus enhancing ATF2-mediated transcription (Figure 1). In addition, *7SK* snRNA sequesters P-TEFb, a general transcription elongation factor and human co-factor for HIV-1 transactivator (Tat) protein, into the catalytically inactive *7SK* snRNP and inhibits HIV transcription [79,80,81,82]. The human T-cell leukemia virus (HTLV)-encoded antisense lncRNA is recruited to the CC chemokine receptor (CCR4) and enhances its transcription to support the proliferation of HTLV-infected cells [169].

Many lncRNAs regulate neighboring genetic loci in a transcript-dependent manner by interfering with the recruitment of transcription factors or Poll II at the promoter, altering chromatin modification or reducing accessibility. LncRNAs can form RNA–DNA triplexes that enrich gene regulatory proteins at the neighboring loci. For example, *PTENpg1* localizes on the promoter of its adjacent locus, *PTEN*, and recruits histone methyl transferases (EZH2 and DNMT3a) to the *PTEN* promoter, dampening *PTEN* transcription [170]. LncRNAs also act as a scaffold for the locus-specific recruitment of chromatin-modifying enzymes. For example, lncRNA *APOAS1* provides a scaffold for the chromatin-modifying histone demethylase protein, LSD1, to localize the *APOA1* gene and repress *APOA1* gene expression [171]. HCV infection induces one such lncRNA that regulates transcription of its neighboring protein-coding gene and alters the antiviral immune response. HCV-infected cells express a nuclear lncRNA *GCSIR* (GPR55 cis-regulatory suppressor of immune response RNA, a.k.a *Lnc-ITM2C-1*) that enhances the transcription of its neighboring gene, *GPR55*, through yet unknown molecular mechanisms. The GPR55 protein, in turn, inhibits the expression of several ISGs, thus, dampening antiviral responses [60].

In addition to regulating transcription, neighboring or intragenic lncRNAs can also modulate the splicing of their protein-coding neighbors. RUNX1 transcription and protein expression are tightly controlled by several lncRNAs transcribed from the neighboring loci. RUNX1 represses HIV-1 replication in T cells by binding to the HIV-1 LTR [172]. RUNX1 gene encodes three transcript variants that produce three different protein isoforms, RUNX1a, b, and c. Among the three, RUNX1b and c have been shown to bind the HIV–LTR and suppress HIV transcription [83]. *LINC01426* (a.k.a. *uc002yug.2*) is transcribed from a locus upstream of *RUNX1*. It enhances the recruitment of splicing factors (MBNL1 and SFRS1) to the regional RNA duplexes resulting in increased *RUNXa* isoform and the relative reduction of *RUNX1b* and c isoform expressions [83]. In addition, *LINC01426* increases the production of HIV protein Tat through unknown mechanisms. Thus, *LINC01426* promotes viral reactivation by inhibiting transcription repressive forms of RUNX1 and enhancing Tat expression [83]. Another lncRNA *RUNXOR* is transcribed from a promoter upstream of the *RUNX1* gene that overlaps with *RUNX1* mRNA. RUNXOR increases H3K4me3 marks at the promoter of *RUNX1* and activates *RUNX1* transcription. Interestingly, myeloid-derived suppressor cells (MDSCs) in people living with HIV (PLWH) showed increased *RUNXOR* expression (Figure 1). An increased expression of *RUNXOR* in MDSCs results in the expression of critical immunosuppressive molecules that cause T cell suppression in PLWH [173].

Although there is an increasing number of lncRNAs identified as novel regulators of host–virus interaction, the precise functional mechanisms of many remain unknown. HCV, IAV, and the Semliki Forest virus (SFV) induce a nuclear lncRNA transcript eosinophil granule ontogeny transcript (*EGOT*). The molecular mechanism of *EGOT*-mediated suppression of the IFN-signaling pathway and enhancement of viral production is yet to be determined [31]. *BISPR* is an IFN-stimulated lncRNA that significantly regulates the antiviral *BST2* gene expression through yet unknown mechanisms [174,175]. IAV-induced lncRNA *TSPOAP1-AS1* inhibits the expression of IFNβ and other ISGs through unknown mechanisms [62]. Interestingly, *TSPOAP1-AS1* is localized in the nucleus and cytoplasm, but IAV-infected cells showed increased nuclear levels of *TSPOAP1-AS1*, implicating a nuclear mechanism of ISG inhibition. ZIKA virus (ZKIV) infection induces lncRNA *OASL-IT1*. *OASL-IT1* enhances IFNβ and ISG (Mx1 and IFITM1) expression and inhibits ZKIV replication through unclear mechanisms [75]. IAV H1N1, H3N2, H7N7 strains, and VSV-infected cells upregulate a nuclear lncRNA *VIN*. The molecular mechanisms of *VIN*-mediated upregulation of viral gene expression remain to be determined [29].

## 4. Heterogeneity in lncRNA Form, Function, and Phenotype

Some lncRNA transcripts utilize diverse mechanisms that occasionally produce contrasting phenotypes highlighting the complexities of their gene regulatory functions. In addition, many lncRNAs are transcribed from genomic regions that encode sequences regulating chromatin structure, which makes investigating molecular mechanisms and interpreting lncRNA functions very challenging in these instances.

This is best exemplified by lncRNA *Ifng-as1*. In mice and humans, *Ifng-as1* [Nettoie Salmonella pas Theiler’s (*NEST*), also named *TMEVPG1*] is transcribed from the opposite strand of the *IFNG* protein-coding region. *Ifng-as1* was initially identified as a susceptibility locus for Theiler’s virus persistence in mice [176]. In follow-up studies, the RNAi-mediated knockdown of human *IFNG–AS1* expression in human T-helper (Th1) cells and significantly reduced *IFNG* transcriptions [177,178]. Similarly, the transgenic expression of mouse *Ifng-as1* enhanced the IFN-γ expression and established resistance to Salmonella enterica and increased persistence of Theilers’ virus in mice [179]. These contrasting phenotypes indicated the essential role of lncRNAs in modulating immune responses to distinct pathogens and disease outcomes. The ectopically expressed *Ifng-as1* transcript binds to a histone methyltransferase complex component (WDR5) and enhances H3K4 trimethylation at the *Ifng* locus in an in vitro cell line model [179]. These approaches indicated that *Ifng-as1* is a trans-acting lncRNA that recruits chromatin modifiers in a sequence-specific manner. A recent study employed CRISPR tools to compare *Ifng-as1* knockout (KO; DNA+RNA product deletion) and *Ifng-as1*-polyA knock-in (KI; truncated non-functional RNA) modification in mice [180]. Deleting DNA (KO) and truncating RNA (KI) inhibited *Ifng* gene expression, but KO mice showed more severe impairment in defense against infection. This study further determined that deleting the *Ifng-as1* locus (KO) eliminates one of the CTCF-binding sites, thus disrupting the chromatin looping required for optimal *Ifng* gene expression. The truncated *Ifng-as1* (KI) does not affect chromatin architecture but diminishes *Ifng* expression. The *Ifng-as1* transcript will likely enhance *Ifng* expression by recruiting and enriching transcription factors or chromatin modifiers. These critical experiments dissecting the effects of the lncRNA transcript and chromatin structure showed that *Ifng-as1* regulates *Ifng* expression in cis, and *Ifng-as1* locus impacts the chromatin organization independent of the Ifng-as1 transcription or lncRNA sequence (Figure 1).

Human *IFNG-AS1* expression was significantly higher in CD4+ Th1 cells, the antigen-specific memory precursor, and the central memory CD8+ T than in the effector memory T cells in LCMV-infected mice. Similarly, the *IFNG–AS1* expression is maintained long term (up to a decade) at high levels in human memory T cells [180] and abundantly expressed in activated Natural Killer (NK) cells [181]. These findings highlight the differences in *IFNG–AS1* expressions in various immune cell phenotypes and indicate its functional relevance in acute and memory responses to viral infections. From a clinical perspective, polymorphisms in the human *IFNG–AS1* gene have been associated with autoimmune and inflammatory disorders [182,183,184], further underscoring their role in immunity and chronic inflammatory diseases in humans.

An IFN-stimulated nuclear lncRNA, *NRIR* (a negative regulator of interferon response; a.k.a. *lncRNA-CMPK2*), produces stimulation-specific contrasting phenotypes. Specifically, *NRIR* inhibited the transcription of several ISGs and enhanced HCV replication in hepatocytes [65]. Moreover, *NRIR* inhibited the expression of IFITM3, a well-characterized ISG, in endothelial and epithelial cells during Hantaan virus infection [185]. However, in monocytes, *NRIR* silencing significantly reduced the LPS-induced expression of ISGs, including MX1, IFITM3, ISG15, and chemokines such as CXCL10. Although these contrasting findings strengthen the role of *NRIR* as a regulator of IFN responses, they highlight the cell-type and stimulus-specific functions of lncRNAs [186].

The lncRNA *NEAT1* sequesters both protein and RNA in the nuclear bodies. An infection with IAV and HSV induces the expression of lncRNA *NEAT1*, which sequesters splicing factor proline glutamine-rich (SFPQ/PSF) to the paraspeckles. SFPQ/PSF acts as a repressor of *IL-8* and HSV viral genes [187], but at the same time, it is essential for IAV mRNA polyadenylation [128]. *NEAT1* activates the antiviral gene *IL-8* transcription by sequestering SFPQ/PSF [187]. However, *NEAT1* also recruits STAT3 to viral gene promoters and upregulates the viral replication in HSV-1 infections [74]. Thus, *NEAT1* functions as an antiviral factor by inducing cytokine response but is simultaneously hijacked by HSV to facilitate viral gene expression. Similar to HSV-1 infection, *NEAT1* upregulation by Hantaan virus (HTNV) infection promotes RIG-I and DDX60 transcription by relocating SFPQ from the promoters of both genes to paraspeckles [56]. Since RIG-I and DDX-60 expression are essential for interferon γ (IFN- γ) production [188,189], it appears that the induction of *NEAT1* enhances antiviral responses against HTNV. During the HIV-1 replication cycle, *NEAT1* sequesters unspliced HIV transcripts in nuclear paraspeckle bodies, thus, preventing the nuclear export of HIV mRNA and promoting the long-term persistence of HIV [51].

Viral infections can also hijack lncRNA functions by manipulating their RBP partners, which are critical for lncRNA activity. For example, HIV integration induces double-stranded breaks (DSB) that initiate the apoptosis pathway in the infected CD4+ T cells. Unlike CD4+ T cells, HIV-infected macrophages have been reported to evade DSB-induced apoptosis by accelerating the decay of *lincRNA-p21*, a lncRNA that inhibits the transcription of pro-survival genes induced during the canonical DNA damage pathway. Two protein binding partners, namely HuR and hnRNP-K, are critical for the stability and function of *lincRNA-p21*. HIV infection of macrophages results in the sequestration of HuR and hnRNP-K in the cytoplasm, where it increases *lincRNA-p21* decay and reduces *lincRNA-p21* levels in the cells. Decreased availability of hnRNP-K in the nucleus reduces the functional nuclear *lncRNA-p21*/hnRNP-K complex required to suppress pro-survival genes. Thus, by sequestering the proteins essential for maintaining *lincRNA-p21* stability and function, HIV inhibits DSB-induced cell death and promotes its persistence in infected macrophages [84]. The expression of lncRNA *SAF* is significantly upregulated in HIV-1-infected human monocyte-derived macrophages (MDM) and HIV-1-infected airway macrophages obtained by the bronchoalveolar lavage of HIV-1-infected individuals. The downregulation of *SAF* increases caspase-3/7 activity levels in virus-infected MDMs, thus inducing apoptosis. Although the mechanisms of *SAF*-mediated regulation of caspase3/7 activity are not completely understood, it is proposed to be a potential target to cause cell death in HIV-infected macrophages and reduce overall HIV burden [190].

Viral lncRNAs have also been shown to manipulate large cellular gene networks through diverse mechanisms. The Epstein–Barr virus (EBV), a tumor-causing virus, is associated with various human cancers [191,192] and encodes noncoding RNAs termed BamHI A rightward transcripts (*BART*s) expressed at high levels in EBV-associated epithelial tumors [93]. *BART*s include microRNAs and lncRNAs [93,192,193]. An alternative splicing of *BART*s results in multiple spliced forms of *BART* lncRNA, with putative open reading frames in *BARF0*, *RK-BARF0*, *RPMS1*, and *A73*, none of which encode proteins [194,195]. *BART* lncRNAs regulate EBV lytic replication [196] and an extensive cellular gene network that influences adhesion, oxidoreductase activity, inflammation, and metastasis [91]. *BART* lncRNAs regulate the expression of tumor suppressor gene *RASA* unfolded protein response (UPR) genes and may contribute to host DNA methylation [91]. High levels of CpG island methylation leading to host gene silencing is associated with EBV-positive gastric carcinomas [197]. *BART* lncRNA significantly inhibits mitochondrial antiviral signaling (MAVS)-induced *IFNB1* promoter activity [92]. Verhoeven et al. [92] observed that *BART* lncRNA *RMS1* is associated with RNA Polymerase II (Pol II) and the CREB-binding protein (CBP/p300) complex in the nucleus. CBP activates transcription by recruiting the transcriptional machinery and also functions as a histone acetyltransferase (HAT) to alter chromatin structure. Thus, *BART* lncRNAs may mediate epigenetic regulation of gene expression through an interaction with the chromatin remodeling complex. They further showed that *BART* lncRNA *RMS1* stalled Pol II at the promoter region of *IFNB1* and inhibited its transcription [92]. Verhoeven et al. showed that *BART* lncRNA *RMS1* expression could inhibit MAVS-induced HAT activity, further indicating that *BART* lncRNA may regulate chromatin remodeling during the gene transcription process in viral infection [92]. An overexpression of *BART* lncRNA *RMS1* also upregulates the transcription of *IKZF3* mRNA, which encodes Aiolos protein normally expressed only in lymphoid cells. Aiolos is expressed in solid and liquid tumors at high levels, promoting tumor cell survival and metastasis [92]. Thus, *BART* lncRNA contributes to viral oncogenesis through multiple mechanisms. In addition, EBV-encoded miRNAs regulated host protein-coding as well as lncRNAs in the EBV-infected cells (reviewed in [198]).

Similarly, human cytomegalovirus (HCMV) encodes at least four known lncRNAs (*RNA1.2, RNA2.7, RNA4.9,* and *RNA 5.0*). *RNA4.9* localizes in the nuclear viral replication complex (VRC) [199], whereas *RNA1.2, RNA2.7*, and *RNA5.0* predominantly localize to the cytoplasm [199,200]. Repressive histone modifications around the major immediate early promoter (MIEP) region inhibit HCMV lytic cycle and latency in myeloid cells [201,202,203,204]. *RNA4.9* is transcribed in latently infected CD14 (+) monocytes and CD34 (+) cells, tethers the components of the PRC complex to the MIEP, enriches the repressive H3K27me3 mark at MIEP, and inhibits viral transcription [90]. *RNA4.9* also mediates the formation of the RNA–DNA hybrid and the initiation of viral DNA replication in the lytic phase [199]. Though *RNA2.7* localizes primarily in the cytoplasm, it has been shown to function in the nucleus and mitochondria. *RNA2.7* binds Polymerase II (PolII) and blocks its interaction with phosphorylated cyclin-dependent kinase (pCDK), thus inhibiting the phosphorylation of Pol II. The inhibition of Pol II phosphorylation leads to host cell cycle arrests and facilitates viral replication [205]. *RNA2.7* interacts directly with the mitochondrial complex protein GRIM-19, prevents its relocalization, and maintains high ATP production levels during lytic infection [206]. In addition, *RNA2.7* is shown to have additional functions with yet unknown molecular mechanisms, such as the inhibition of apoptosis and maintenance of latency by the suppression of lytic gene expression in latent cells [207]. *RNA2.7* stabilizes cellular transcripts that promote cellular motility and viral spread in lytic infection [208]. *RNA1.2* is expressed at high levels during lytic infection, inhibits cellular NF-kB activation, and mediates the extracellular release of IL-6 [209].

Herpes simplex virus (HSV)-encoded Latency-associated transcript (*LAT*) is the only viral transcript expressed during the latent infection of neurons and plays an important role in HSV latency. *LAT* long non-coding transcript accumulates in the nucleus and contributes to the silencing of viral lytic genes by the heterochromatization of their promoters. *LAT* is suspected to be involved in the recruitment of chromatin remodeling complexes during heterochomatization, although the mechanisms are unclear [210,211,212,213,214]. In addition, the *LAT* transcript encodes microRNA (miRNA), small RNA (sRNA), short non-coding RNA (sncRNA), and open reading frame (ORF) encoding proteins that mediate numerous functions to maintain latency. The functions of *LAT*-encoded miRNAs, sRNAs, sncRNAs, and ORFs have been studied extensively and reviewed previously [215,216].

Numerous studies have revealed that while some lncRNAs affect multiple viral infections, several virus-induced lncRNAs act independently or in concert to regulate a single pathway. Although our understanding of lncRNA function in viral pathogenesis has significantly improved, future studies should focus on identifying and characterizing lncRNAs that have pronounced effects on virus replication, infection outcomes, and, more importantly, their molecular mechanisms. Investigating the mechanisms of lncRNA functions has immense therapeutic potential, as lncRNAs could serve as molecular targets for future antiviral therapy.

## 5. Therapeutic Potential of lncRNAs

Given their proven role in cellular defense and viral pathogenesis, careful functional studies are needed to define the diagnostic or therapeutic potential of lncRNAs. While most research focuses on understanding lncRNA functional biology, recent studies also explored their potential diagnostic and therapeutic targets [217]. Most studies described in this review have used lncRNA knockdown or overexpression to decipher the functional impact of lncRNAs on the expression of their protein-coding targets, viral replication, and immune response. A diverse array of modalities, such as RNA interference using si/shRNA [218], antisense oligonucleotides (ASO) [219], CRISPR-mediated knockout (CRISPR-KO), knock-in (CRISPR-KI) [180], transcriptional activation or inhibition (CRISPRi/a), and CRISPR-mediated RNA silencing [220,221,222,223] are available to manipulate lncRNA expression for functional studies (Table 2). In addition, various strategies have been employed to augment lncRNA expression in the local genomic context. Talen-mediated knock-in of strong promoters upstream of lncRNA transcription start site [224] and transcriptional activation using CRISPR/dCas9 (CRISPRa) [225] have been used to activate lncRNA expression from their endogenous loci. CRISPR-display is a compelling strategy that allows for the site-specific delivery of lncRNA transcripts using CRISPR/dCas9 and guide the RNA sequence fused with the lncRNA sequence [226]. Small-scale lncRNA knockdown screens have yielded significant insights into the role of virus-induced lncRNAs on viral pathogenesis [77,227]. Many experimental methods are used to probe lncRNA interactions with their protein-binding partner or chromatin and investigate lncRNA functions. However, most methods are technically challenging, need skilled researchers, specialized equipment, and reagents, and, thus, are prohibitively expensive for most laboratories. Several computational methods are being developed to predict lncRNA interactions and functions systematically. For example, lncRNA–DNA interactions that may regulate transcription could be predicted using computational methods such as Triplexator [228,229], Triplex Domain Finder [230], LongTarget [231], and Triple [232,233], which examine whether lncRNAs form triplexes with target promoters and enhancers. “Super-lncRNAs” [234] predict lncRNAs that bind super-enhancers through triplex formation. However, the functional impact of lncRNAs on virus infection must be rigorously investigated using genome-wide functional studies in cellular models and primary cells.

The thorough investigation of lncRNA in tumor biology [235] using preclinical models led to the development of lncRNA-based diagnostic [236] and therapeutic modalities for cancer, with some showing promising results in human clinical trials [237]. LncRNAs that show the potential to enforce viral latency or reactivation could be good candidates for direct therapeutic targeting. The epigenetic modulation of viral latency using latency reversal agents such as HDAC inhibitors can affect a wide gene network and show off-target effects. Recruiting or deterring an lncRNA to or from a virus promoter could provide specificity in altering viral latency. An interaction with a specific RNA-binding protein partner is essential to lncRNA function; accordingly, small molecule therapeutics may be used directly to disrupt the critical interactions [84,238]. Computational tools are being researched and developed to use nucleotide sequences and structural motifs of lncRNAs to predict their subcellular localization [239,240,241,242], interactions with RNA-binding proteins, and functions. However, successful therapeutic targeting of lncRNAs will depend on our ability to precisely identify relevant RNA motif/s and better understand the structural and functional features of lncRNAs.

## 6. Conclusions

In recent years, we have seen an avalanche of new information on lncRNA expression in virus-infected cells and the identification of several lncRNAs affecting viral replication and host immune responses, all of which have improved our understanding of the diverse functional potential of lncRNAs. This is a burgeoning area of research beyond mammalian species [243]. Some aspects of lncRNAs have not yet been studied in infectious disease research. For example, cellular stress, such as DNA damage [244,245], rapamycin treatment [246], and cellular differentiation [247], regulate the subcellular localization of lncRNAs. A recent study showed that an influenza virus-induced murine lncRNA, Lnc45, resides mainly in the nucleus in uninfected cells but translocates to the cytoplasm in H5N1-infected cells and dramatically impedes viral replication [248]. This observation highlights the need for a systematic study of the cellular redistribution of lncRNAs in viral infections. Some lncRNAs are highly conserved across species, but most show lower sequence conservation than protein-coding genes [249,250,251]. Therefore, it is necessary to examine the sequence and functions of relevant lncRNAs in higher animal models that are closer to humans like non-human primate (NHP) species and evaluate the use of NHP models in preclinical studies for the therapeutic targeting of lncRNAs. The systemic or specific delivery of lncRNAs or lncRNA-inhibiting RNA-based therapeutics is currently under investigation for the clinical management of several human diseases [252]. Optimal delivery modalities to target specific cells and tissues are being intensely studied. An in-depth analysis of lncRNAs in viral infections, particularly, those establishing latent reservoirs, such as HIV infection, has enormous potential for discovering novel regulatory mechanisms associated with immune response/inflammation, viral replication, and long-term viral persistence. These studies can potentially lead to identifying novel and highly selective therapeutic targets.

## Figures and Tables

**Table 1:** Cellular and viral nuclear lncRNAs regulate viral replication and persistence.

LncRNA	Virus	Mechanism	Reference
Proviral lncRNAs			
*NRAV*	IAV, SeV, MDRV, HSV	Histone modification and reduction in active transcription marks at ISG.	Ouyang et al., 2014 [57]
*TSPOAP1-AS1*	IAV	*TSPOAP1-AS1* inhibits FNβ1 transcription, ISRE activation, and ISG expression.	Wang et al., 2019 [62]
*Lnc-MxA*	IAV	Lnc-MxA inhibits IFNβ transcription by binding to its promoter and enhances viral replication.	Li et al., 2019 [61]
*VIN*	IAV, VSV	*VIN* increases virus replication and viral gene expression. Molecular mechanisms are unknown.	Winterling et al., 2014 [29]
*EGOT*	HCV, IAV, SFV	*EGOT* inhibits the expression of several ISGs and enhances viral replication. Molecular mechanisms are unknown.	Carnero et al., 2016 [31]
*Lethe*	HCV	*Lethe* inhibits RelA-mediated DNA-binding; inhibits expression of antiviral factors, protein kinase R (PKR), 2′,5′-oligoadenylate synthetase (OAS) proteins, and Interferon Regulatory Factor 1 (IRF1), and enhances HCV replication	Rapicavoli et al., 2013 [66]; Xiong et al., 2015 [71]
*LncRNA RP11- 288L9.4*	HCV	*TSPOAP1-AS1* inhibits expression of IFNα-inducible protein 6 (IFI6) by histone modification and enhances HCV replication.	Liu et al., 2019 [72]
*NRIR*	HCV	*NRIR* inhibits transcription of several interferon-stimulated genes (ISG) and enhances HCV replication.	Kambara et al., 2014 [65]
*Lnc_000641*	pseudorabies virus (PRV)	*Lnc_000641* inhibits IFNα transcription, phosphorylation of transcription factors (Jak and STAT1), and increases PRV replication.	Fang et al., 2021 [73]
*NEAT1*	HSV-1	*NEAT1* recruits STAT3 to viral gene promoters to increase viral gene expression.	Wang et al., 2017 [74]
**Antiviral lncRNA**			
*IVRPIE*	IAV	*IVRPIE* upregulates IFNβ and several ISGs, including IRF1, IFIT1, IFIT3, Mx1, ISG15, and IFI44L, by affecting histone modification of these genes.	Zhao et al., 2020 [63]
*OASL-IT1*	ZKIV	*OASL-IT1* enhances expression of IFN-β, Mx1, IFITM1 and inhibits ZKIV replication.	Wang et al., 2021 [75]
*LUARIS*	EMCV, HBV, HCV	*LUARIS* upregulated the level of IFN-stimulated genes through interactions with hnRNPU and ATF2 and suppressed EMCV, HBV, and HCV.	Nishitsuji et al., 2016 [76]
*NEAT1*	Hantaan virus	*NEAT1* relocates SFPQ to paraspeckles, increases RIG-I and DDX60 transcription, increases IFN- γ, and inhibits virus.	Ma et al., 2017 [56]
**LncRNAs influence the long-term persistence of the virus**			
*NRON*	HIV	*NRON* mediates degradation of HIV Tat protein.	Li et al., 2016 [77]
*MALAT1*	HIV	*MALAT1* promotes HIV reactivation from latent provirus.	Qu et al., 2019 [78]
*7SK*	HIV	*7SK* promotes HIV latency by inactivating p-TEFb.	Nguyen et al., 2001 [79] Contreras et al., 2007 [80]; Budhiraja et al.,2013 [81]; Eilebrecht et al., 2017 [82]
*uc002yug.2*	HIV	*uc002yug.2* promotes viral reactivation by inhibition of Transcription Repressor RUNX1.	Huan et al., 2018 [83]
*lincRNA-p21*	HIV	*lincRNA-p21* inhibits DSB-induced cell death, promotes viral persistence.	Barichievy et al., 2018 [84]
*HEAL*	HIV	*HEAL* promotes viral reactivation by recruiting histone acetyltransferase p300 to HIV-1 promoter region.	Chao et al.,2019 [85]
*NEAT1*	HIV	*NEAT1* sequesters unspliced HIV transcripts in nuclear paraspeckle bodies promoting long-term persistence of HIV.	Zhang et al., 2013 [51]
*HIV antisense lncRNA*	HIV	*HIV antisense lncRNA* recruits chromatin remodeling proteins such as DNMT3a, the enhancer of Zeste 2 (EZH2), and histone deacetylase 1 (HDAC-1) to HIV 5′long terminal repeat. These proteins bring about H3K9 dimethylation, H3K27 trimethylation, and histone deacetylation, resulting in epigenetic silencing of viral transcription.	Saayman et al., 2014 [86]
KSHV-encoded *PAN RNA*	KSHV	*PAN* RNA binds lysine demethylases UTX and JMJD3, and the lysine methyltransferase MLL2 facilitates the recruitment of histone demethylases to the viral chromatin.	Rossetto et al., 2012 [87] Rossetto, 2013 [88] Rossetto, 2016 [89]
HCMV-encoded *RNA4.9*	HCMV	*RNA4.9* tethers the components of the polycomb repression complex (PRC) to the major immediate early promoter region (MIEP) and represses viral transcription.	Rosseto., 2013 [90]
EBV-encoded *BART lncRNA*	EBV-associated epithelial tumors	*BART* lncRNAs downregulate the expression of the tumor suppressor gene RASA1 and unfolded protein response (UPR) genes. BART lncRNAs regulate host gene expressions through chromatin modification.	Marquitz.,2015 [91]; Verhoeven, 2019 [92]
EBV-encoded lncRNA *BHLF1*	EBV-associated epithelial tumors	*BHLF1* localizes at the surface of the viral replication compartment and forms an RNA–DNA hybrid at the site of virus transcription.	Park & Miller, 2018 [93]; Rennekamp & Lieberman, 2011 [94]

**Table 2:** Methods to modulate lncRNA expression for functional studies.

Method	Use	Advantages	Limitation
siRNA/shRNA	Knockdown	Inexpensive, cost-effective for large-scale screening	Nuclear lncRNAs cannot be targeted efficiently by siRNA; structural constraints limit accessibility, large-scale off-target cleavage, and knockdown may be short-lived.
Antisense Oligo (ASO)	Knockdown	Efficient degradation of nuclear lncRNA	Structural constraints limit accessibility, large-scale off-target cleavage, and knockdown may be short-lived.
CRISPR/Cas9	Gene knockout or knock-in	Easily programmable to target genes of interest, most definitive	CRISPR/Cas9-mediated frameshift mutations are not helpful for most lncRNAs as their functional sequence motifs are unknown. CRISPR/Cas9 excision of the entire lncRNA gene may disrupt overlapping coding or noncoding RNA region.
CRISPRi	Inhibition of transcription	Easily programmable to target genes of interest	CRISPRi may deregulate overlapping coding or noncoding RNA region, the functions of lncRNA transcript from those of promoter or enhancer element encoded within the lncRNA locus or small peptide encoded by the transcript.
CRISPR/Cas13d	Knockdown	Easily programmable, independent of PAM, superior RNA knockdown efficiency and dramatically higher specificity than currently available methods, stable long-term expression	Cannot decipher the function of enhancer element encoded within the lncRNA locus or small peptide encoded by the transcript
CRISPRa	Activation of transcription	Easily programmable, enhanced lncRNA expression from the endogenous loci	Dependence on protospacer-adjacent motif (PAM); may deregulate overlapping coding or noncoding RNA region; and the functions of lncRNA transcript cannot be distinguished from those of promoter or enhancer element encoded within the lncRNA locus or small peptide encoded by the transcript.
CRISPR-display		Easily programmable, allows site-specific delivery of lncRNA transcript to desired genomic loci; this method can be used to test both *cis* and *trans* effects of lncRNA transcripts and distinguish them from the act of lncRNA transcription.	Limited by the number of available functional RNA motifs and RNA-binding protein functions
